# Effects of furosemide and tadalafil in both conventional and nanoforms against adenine-induced chronic renal failure in rats

**DOI:** 10.1186/s40001-022-00747-3

**Published:** 2022-07-11

**Authors:** Moustafa Mahmoud Hamdy, Mahran S. Abdel-Rahman, Dalia M. Badary, Mahmoud S. Sabra

**Affiliations:** 1 Pharmacology Department, Faculty of Medicine, Assiut University, Assuit, 71526 Egypt; 2 Pharmacology and Toxicology Department, Faculty of Pharmacy, Sphinx University, New Assuit, 71526 Egypt; 3 Pathology Department, Faculty of Medicine, Assiut University, Egypt, Assuit, 71526 Egypt; 4 Pharmacology Department, Faculty of Veterinary Medicine, Assiut University, Assuit, 71526 Egypt

**Keywords:** Adenine–CRF in rats, Tadalafil, Furosemide, Nanoparticles, NGAL, Caspase-3, IL-1β

## Abstract

**Background:**

Chronic renal failure (CRF) is a progressive loss of renal function that lead to reduced sodium filtration and inappropriate suppression of tubular reabsorption that ultimately leads to volume expansion. The aim of this study was to study the efficacy of furosemide and tadalafil nanoforms compared to conventional forms against adenine-induced CRF rat-model.

**Methods:**

Addition of 0.75% adenine to the diet of rats for 4 weeks gained general acceptance as a model to study kidney damage as this intervention mimicked most of the structural and functional changes seen in human chronic kidney disease Urine analysis, histopathological changes and immunohistochemical expression of caspase-3 and interleukin-1 beta (IL-1β) in renal tissues were performed.

**Results:**

Our results showed that the combination of tadalafil and furosemide using conventional and nanoparticle formulations had better renoprotective effect than individual drugs. This was demonstrated by improvement of urinary, serum and renal tissue markers as indicative of organ damage. This was also reflected on the reduction of tubular expression of kidney injury molecule-1 (KIM-1) and neutrophil gelatinase-associated lipocalin (NGAL).

Immunohistochemical studies showed that the deteriorated renal cellular changes indicated by increased expression of caspase-3 and IL-1β were greatly improved by the combined treatment particularly with the nanoforms.

**Conclusions:**

The nanoforms of both furosemide and tadalafil had greater renopreventive effects compared with conventional forms against adenine-induced CRF in rats.

**Supplementary Information:**

The online version contains supplementary material available at 10.1186/s40001-022-00747-3.

## Background

Chronic renal failure (CRF) is an international and national health problem that increases the risk of mortality and the use of specialized health care. Chronic renal failure is characterized by progressive loss of renal function that lead to reduced sodium filtration and inappropriate suppression of tubular reabsorption that ultimately leads to volume expansion. Chronic renal failure is also associated with inflammation and oxidative stress leading to endothelial dysfunction, glomerular fibrosis, and mesangial expansion. Fluid overload frequently occurs in patients with moderate to particularly late stages of CRF and has been associated with hypertension, congestive heart failure, left ventricular hypertrophy as well as edema [1].

Phosphodiesterase-5 (PDE5) inhibitors were originally developed to treat angina pectoris. However, it is subsequently used for erectile dysfunction and pulmonary hypertension [2]. There is an increasing evidence suggests that PDE5 inhibitors including sildenafil, vardenafil, and tadalafil have broader effects, most likely due to their ability to inhibit the breakdown of cyclic 3′, 5′-guanosine monophosphate, the second messenger for nitric oxide (NO) and natriuretic peptides [3].

Previous studies have demonstrated that PDE5 inhibitors improve endothelial function and possess nephroprotective effects in renal ischemia–reperfusion injury [4, 5]. In addition to their vasodilatory action, PDE5 inhibitors possess anti-apoptotic and anti-oxidant properties, making them a promising therapy for ischemia–reperfusion injury of various organs [6]. Tadalafil 5–20 mg was well tolerated in patient with end-stage renal failure undergoing haemodialysis. Furthermore, chronic kidney disease resulted in significant reduction of cytochrome 3A2 expression and activities in rats. Increased systemic exposure of numerous cytochrome 3A4-substrate drugs, as tadalafil, was established in patients with CRF [7, 8].

Chronic administration of PDE5 inhibitors could attenuate renal injury and the increased blood pressure in animal models of diabetic nephropathy, renal ischemia‐reperfusion injury, and CRF [9–11]. Furthermore, the inhibition of nitric oxide/cyclic guanosine monophosphate signaling in the renal tissue can retard renal function. On the other side, PDE5 inhibitors cause vascular smooth muscle relaxation, and consequently, decreasing blood pressure. Therefore, they may be new effective treatments for renal failure, as they increase cyclic guanosine monophosphate levels [12].

Loop diuretics were traditionally used to enhance renal excretion of excess salt and water. Blockage of sodium–potassium–chloride cotransporter in the thick ascending limb of the loop of Henle by the loop diuretics decreases cellular transport and reduces energy consumption and, therefore, preserves cellular vitality. Loop diuretics should be kept for conditions of clinically significant fluid overload such as heart failure and significant fluid retention or with advanced kidney failure and can be combined with thiazide‐ diuretics [13]. Studies revealed that furosemide could inhibit carbonic anhydrase enzyme by acting on zinc-binding group of carbonic anhydrase enzyme. In addition, furosemide could inhibit different isoforms of carbonic anhydrase I, II, and XI [14, 15]. The inhibition of carbonic anhydrase I by furosemide has been conveyed to cause vasodilation and a decrease in blood pressure [16]. Various studies demonstrated the usefulness of furosemide in different chronic kidney diseases [13, 17].

Rat model of reduced renal mass-induced CRF is characterized by a decreased sodium–chloride reabsorption and fluid from the proximal renal tubules leading to improved delivery and reabsorption in the loop of Henle, distal tubule, and collecting ducts. Rat models of reduced renal mass showed a consistent reduction in the expression of the sodium-transporting proteins and the sodium/potassium adenosine triphosphatase, but a relative three to fourfold increase in the expression of the protein for the bumetanide-sensitive sodium–potassium–chloride cotransporter-1 transporter in the cells of the thick ascending limb of the loop of Henle in the remaining or residual nephrons. These are the respective targets for loop diuretics. The increased level of fluid delivery and reabsorption in loop of Henle, together with the relative maintenance of the target carriers per nephron in the loop of Henle, is a dependent factor in the reserved efficacy of loop diuretics even in patients with chronic renal failure [18, 19].

The half-time of furosemide ranges from 0.5 to 2 h, but can be extended in renal failure. Approximately half of the administered dose of furosemide is metabolized in kidney to the glucuronide. The remainder is eliminated by active renal process. The unmetabolized and secreted drug fraction can inhibit sodium–chloride reabsorption from the thick ascending limb of the loop of Henle. In patients with CRF, the elimination of furosemide, is greatly delayed, so prolonging its actions and decreasing any differences in the array of response to these drugs [16].

Patients with CRF manifest diuretic resistance. Reduced basal level of sodium reabsorption, and enhanced sodium–chloride reabsorption in distal segments, combined with a decreased delivery of diuretic to the kidney could limit diuretic responsiveness in patients with CRF. One critical factor that limits the delivery of diuretics to their renal sites in patients with CRF is a reduction in renal blood flow. Therefore, a decrease in renal blood flow in patients with CRF reduces both the efficacy and metabolism of furosemide. The reduced responsiveness requires an increase in dose. The increase in dose combined with the reduction in metabolism leads to an increase in furosemide plasma levels in patients with CRF and consequent serious ototoxicity in these patients [20].

These renal targeting drugs will increase the efficacy and reduce the toxicity of new, established, and pre-existing drugs. The use of bionanotechnology in therapeutics of kidney diseases have been developed recently on polymer-based nanometers, which have great attention in the field of drug delivery applications [21]. The significant advantages of nanoparticles used as drug carriers compared to conventional forms are high stability, high carrier capacity, possibility of integration of both hydrophilic and hydrophobic drugs, and probability of variable routes of administration [22]. Numerous polymers could efficiently bring the drugs in the optimum dosage to the target site so increases the therapeutic effects while diminishing side effects [23]. To improve loop diuretic delivery to renal tubules, to overcome diuretic resistance, and to increase renal blood flow in rat model of CRF we combined tadalafil as vasodilator PDE5 inhibitors with furosemide loop diuretic. In addition, we expected that enhanced drug delivery would be associated with nanoparticulation of the chosen drugs. To the best of our knowledge, there is no available studies about furosemide and tadalafil drug interactions in chronic renal failure models.

Based on the previous information, to the best of our knowledge, no previous studies were conducted to evaluate the possible role of combined administration of PDE5 inhibitor and loop diuretic, particularly in nanoformulations in chronic renal failure induced chemically. Therefore, this study was aimed to evaluate the renoprotective effects of tadalafil and/or furosemide loaded and unloaded in nanoparticles in adenine-induced CRF in rats.

## Materials and methods

### Animals and induction of chronic renal failure (CRF)

The experimental protocol was approved by the Institutional Animal Care & Use Committee (IACUC) of the Faculty of Medicine, Assiut University, Assiut, Egypt (approval number: 17200705). The experiment was conducted using adult male albino rats (8–10 weeks) weighing 150–250 g. The animals were housed in the animal house of the Faculty of Medicine, Assiut University under standard laboratory conditions and maintained under natural light and dark cycle with free access to food and water. Animals were randomly assigned to the experimental groups, 6–8 animals each. Chronic renal failure (CRF) was induced by addition of 0.75% w/w adenine to the diet of rats for 4 weeks [1].

### Experimental design

Animals were randomly divided into 9 groups; 6–8 rats each. The rats were orally administered with tadalafil (5 mg/kg p.o.) according to the previous studies [24–26]. The rats were intramuscularly administered with furosemide (20 mg/kg i.m.) according to the previous study [27]. Group 1 kept as control group in which rats were given saline. Group-2 received adenine to induce CRF and kept as CRF group. Group-3 received adenine plus plain tadalafil (5 mg/kg p.o.) dissolved in saline. Group-4 received adenine plus plain furosemide (20 mg/kg i.m.) for 28 days. Group-5 received adenine plus combination of plain tadalafil (5 mg/kg p.o.) and plain furosemide (20 mg/kg i.m.). Group-6 received adenine plus tadalafil-loaded nanoparticles (NPs). Group-7 received adenine plus furosemide-loaded NPs. Group-8 received adenine plus the combination of tadalafil and furosemide-loaded NPs. Group-9 animals with carrier-based NPs (chitosan and poly lactic-co-glycolic acid (PLGA)).

### Preparation of chitosan (CS)/alginate (ALG) nanoparticles loaded with furosemide

Furosemide loading NPs were kindly provided by the National Research Center (NRC), Cairo, Egypt. The optimum NPs preparation procedures were performed according to Radwan et al. [28] and as follows: The pH of 10 mL ALG solution (300 mg/100 mL) was modified to pH 5.1 by the addition of 0.5 M HCl. A calculated amount of CS was dissolved in 1% acetic acid solution overnight followed by sonication for 10 min. The pH of CS solution was adjusted to 5.4 using 2.5 M NaOH solution. Two mL calcium chloride (CaCl2) solution (332 mg/100 mL) were added drop wise, at a rate of 1 mL/min to 10 mL ALG solution while stirring by a magnetic stirrer at 480 rpm for 30 min. Four mL CS solution of 80, 160, 240 mg/100 mL were then added dropwise to the calcium ALG pre-gel and stirring was continued for an additional 1 h. The formed NPs were centrifuged using high speed cooling centrifuge (Sigma 30 K, Osterode am Harz, Germany) at 14,000 rpm at 4 °C for 30 min. The supernatant was removed and the precipitate was washed and reconstituted in 15 mL filtered distilled water and sonicated for 10 min. The NPs suspension was frozen for 24 h at − 30 °C, then dried in a laboratory freeze-dryer. For drug loading, one mL of furosemide in ethanol containing various amounts of the drug (5, 10, 20, 40 mg), was incorporated into the ALG solution and sonicated for 1 min before adding the CaCl2 solution [28].

### Preparation of poly lactic-co-glycolic acid (PLGA) nanoparticles loaded with tadalafil

Tadalafil loading NPs were kindly provided by the National Research Center (NRC), Cairo, Egypt. Tadalafil-loaded NPs were prepared according to the solid-in-oil-in-water (s/o/w) emulsion technique [29]. PLGA (35 mg) was dissolved in dichloro-methane for 6 h to obtain a uniform PLGA solution. Normal Tadalafil 15 mg was added to the PLGA solution and sonicated at 55 W for 1 min to produce the solid-in-oil primary emulsion. This emulsion was added to 20 ml of polyvinyl alcohol solution (1% w/v) and again sonicated at 55 W for 2 min to get the final solid-in-oil-in-water emulsion. The resulted nano-sized particles were stirred in the emulsion for 3 h for solvent evaporation. The final emulsion was centrifuged at 15,000 rpm for 15 min to remove the residual solvent. The NPs obtained were washed thrice with deionized distilled water, and finally resuspended in deionized water and dried on a lyophilizer. The NPs were stored at 4 °C till further use [29].

### Validation of nanoparticles by Transmission Electron Microscopy (TEM)

The size and morphology of the NPs were evaluated using a transmission electron microscope (TEM) JEM-2100 HR (Jeol, USA) by high resolution TME at an accelerating voltage of 200 kV at National Research Center (NRC), Egypt. The lyophilized drug-NPs solution (1 mg/mL) were placed on copper grids covered with nitrocellulose membrane and stained with 1% (w/v) sodium phosphotungstate solution. About 15 min after NPs deposition, the grid was then loaded into TEM, and the size and morphology were assessed [30].

### Measurement of Zeta potential

The measurement of Zeta potential was performed using Zeta Potential Analyzer (National Research Center, Egypt) according to Sivakumar et al. [31]. The measurement of Zeta potential was performed in double distilled water using disposable Zeta cells and the standard protocol at 25 °C. The instrument was calibrated routinely with a − 50 mV latex standard. The mean zeta potential was determined using phase analysis light scattering technique as previously described [32].

### Determination of urine volume and fluid intake

Twenty-four hour urine collection by metabolic cage twice at the first and fourth week during the induction of CRF. The calculation of urine volume, fluid intake and urine analysis were then performed according to a prior study [33].

### Determination of urinary albumin

Albumin was determined using available commercial kit (Cat#DIAG-250-BioAssay Systems-U.S.A) followed the standard protocol. The intensity of the color measured at 620 nm which is directly proportional to the albumin concentration in the sample as described [34].

### Determination of urinary glucose

Glucose was determined using available commercial kit (Cat# EGL3-100-BioAssay Systems-U.S.A) following the manufacturer’s instructions. The intensity of the color was measured at 560 nm which is directly proportional to the glucose concentration in the sample as reported [35].

### Determination of urinary ketone bodies

Ketone bodies were determined using available assay kit (Cat# EKBD-100-BioAssay Systems-U.S.A) followed the standard protocol. The intensity of the color was measured at 340 nm which is directly proportional to acetoacetic acid and 3-hydroxybutyric acid concentrations in the sample [36].

### Determination of urine osmolarity

Urine osmolarity (Uosm) was measured using osmometer (Osmette A) and according to El-Shabrawy et al*.* [37].

### Determination of specific gravity of the urine

Urine specific gravity was measured using a reagent strips for rapid detection of specific gravity according to a previous study [38].

### Assessment of renal functions

Renal functions were monitored by measuring serum creatinine (Cat. no. 234-000), blood urea nitrogen (Cat. no. UR 21-10) and total protein (Cat. no. 310-001) using commercially available assay kits (Schiffgraben, Hannover, Germany) according to the manufacturer’s instruction. The previous parameters are measured spectrophotometrically.

### Markers of the oxidative stress

Malondialdehyde was measured spectrophotometrically in kidney tissue homogenates using available commercial kits (Schiffgraben, Hannover, Germany) (Cat. no. MD 25–28) according to a previous study [39], nitric oxide (NO) contents (Cat. no. NO 25–33) as previously reported [40] and reduced glutathione (GSH) (Cat. no. GR 25–11) as described [41].

### Selective biomarkers for evaluating chronic renal failure

Kidney injury molecule-1 (KIM-1) which is a type-1 transmembrane protein, is not normally present, but is expressed on the proximal tubule apical membrane in rodent kidneys after renal injury. KIM-1 was assayed by available commercial ELISA kit (Cat. no. E-EL-R3019, Sunlong Biotechnology, Shangyi, Hangzhou, Zhejiang, China) following the manufacturer’s instructions and as reported [42]. Neutrophil gelatinase-associated lipocalin (NGAL) which is a member of the lipocalin superfamily that is highly expressed in the rodent kidneys following injury especially at proximal convoluted tubule. NGAL was assayed by available commercial ELISA kit (Cat. no. E-EL-R0662, Sunlong Biotechnology, Shangyi, Hangzhou, Zhejiang, China) [43].

### Histopathological and immunohistochemical studies

Kidney tissues were fixed in 10% formalin for 24 h followed by dehydration and embedded in paraffin. About 4 µm-thick kidney sections were sectioned and stained with hematoxylin–eosin (H&E). Light microscopic analysis was performed in 20 randomly selected areas in each section by blinded observation. The histopathological examination was performed to determine the extent of tubulointerstitial tissue and glomerular alterations. The tubulointerstitial damage involved tubular necrosis, atrophy, lumen dilation and inflammatory cell infiltration. Tubular damaged was scored on a scale from 0 to 4 (no necrosis cored 0, focal necrotic areas of ≤ 25% of the kidney scored 1, necrotic area was about 26–50% of kidney scored 2, necrotic area was 51–75% of kidney scored 3 and with the necrotic area forming about 76–100% of kidney scored 4). The average score was used for comparison according to a prior study [44].

Interleukin-1 beta (IL-1β) and Caspase-3 were analyzed by immunohistochemical staining. Tissue sections (4-µm thick) were deparaffinized in xylene and rehydrated. The sections were immersed in 3% H_2_O_2_ for 10 min to eliminate endogenous peroxidase activity then washed with PBS (2 min × 3 times). They were then incubated with normal goat serum according to the standard manufacturing protocol (Vector Laboratories, Burlingame, CA) at 37 °C for 30 min, after which the sections were incubated with primary polyclonal rabbit active anti-caspase-3 antibody in dilution 1/200 (E-AB-6602, Elabscience Biotechnology inc, USA) and polyclonal rabbit anti-IL-1 β antibody; 1/100 (E-AB-66749, Elabscience Biotechnology inc, USA) for 1 h at room temperature. Polyperoxidase–anti-Mouse/Rabbit IgG was then added for 20 min. The antigen–antibody complex was detected using a streptavidin–biotin–peroxidase kit and counterstained with Mayer’s hematoxylin. Positive and negative control sections were used for each assay.

The active caspase-3, and IL-1beta immunostaining cells were identified by intense brown nuclear and cytoplasmic staining. The immunoreactivity of caspase-3, and IL-1beta was described as a histological score (H-SCORE) which obtained by multiplying the number of activated cells (0–100% of cells) by the intensity of staining (1 = weak, 2 = moderate, 3 = strong). The sections were examined using light microscope (Olympus BX41, New York) at high magnification (400×) in the 20 randomly selected areas. Blinded fashion assessment was performed and the average score of all groups was used for comparisons. Photomicrographs were taken using digital camera (ToupCam LCMOS05100KPA).

### Statistical analysis

Data for each measured parameter were tested for the normality of distributions (Shapiro–Wilk test, *p* > 0.05). Statistical significance was assessed by one way ANOVA for repeated-measures, or two-way ANOVA as appropriate. The Dunnett test and Tukey’s multiple comparisons test were used for data point comparisons in each group. Data are presented as means ± SEM. Data of *p* ≤ 0.05 was considered statistically significant. Graph Pad prism® software (version 8) was used to performed these statistical analyses.

## Results

### Test the normality of distributions

Data were analysed for normal distributions using the Shapiro–Wilk test, the most potent normality test, compared to the other tests such Kolmogorov–Smirnov, lilliefors and Anderson–Darling tests**.** The Significance values of the Shapiro–Wilk test for measured parameters were greater than 0.05. Thus, the data of the present study were normal.

### Physicochemical characteristics of furosemide- and tadalafil-loaded nanoparticles

**Fig. 1:**
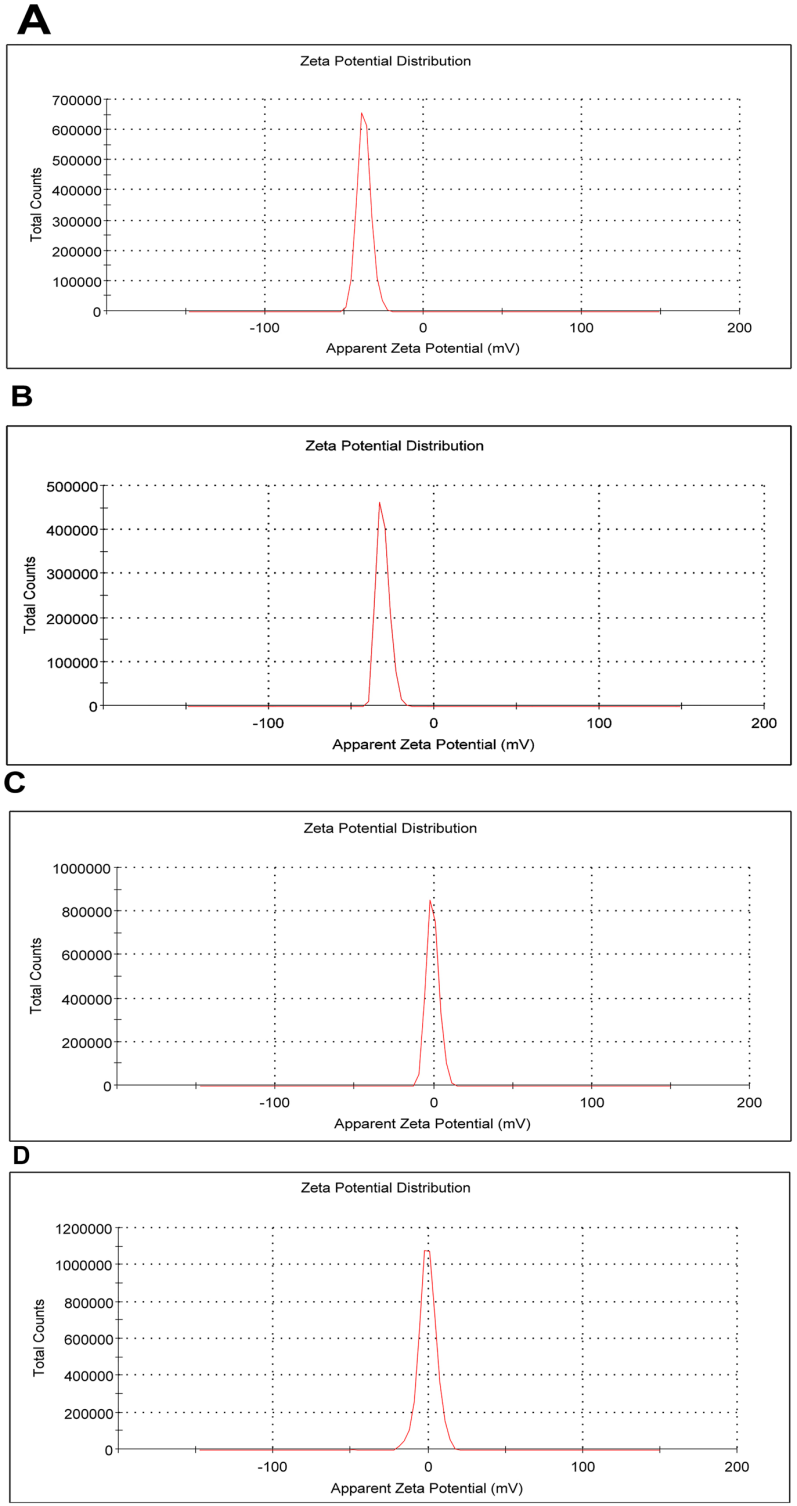
Zeta potential distribution of chitosan/alginate nanoparticles (**A**) and furosemide loading in chitosan/alginate nanoparticles (**B**) & zeta potential distribution of poly lactic-co-glycolic acid nanoparticles (**C**) and tadalafil loading in poly lactic-co-glycolic nanoparticles (**D**)

**Fig. 2:**
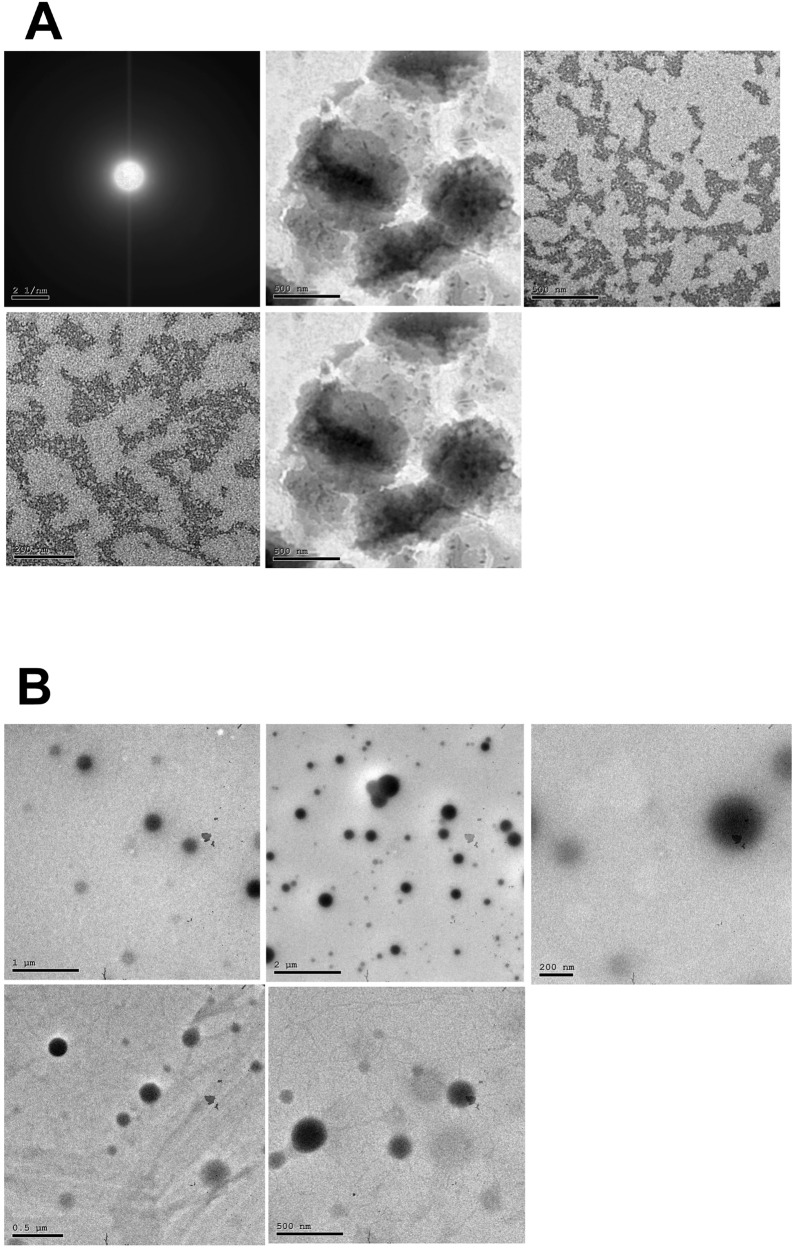
Transmission electron microscopy photomicrographs depicting the spheroidal forms of furosemide nanoparticles (**A**) and the regular spherical shape of tadalafil nanoparticles (**B**) at different magnifications

Furosemide-loaded NPs were prepared as previously reported [28] and described in the methods. In the present study, the nanomaterial chitosan/alginate was employed. As validated by TEM, Furosemide-loaded NPs are less than 50 nm in diameter with a spheroidal shape and suspension form (Fig. 2A). As measured by Zeta Potential Analyzer, the charge density of chitosan/alginate NPs found to be − 37 Mv (Fig. 1A) and the charge dropped to − 31 Mv (Fig. 1B) upon loading of furosemide into chitosan/alginate. Tadalafil-loaded NPs were prepared as described by [29]. As shown by TEM photomicrograph, tadalafil-loaded NPs are 200 ± 50 nm in diameter with a regular spherical shape and suspension form (Fig. 2B). As measured by Zeta Potential Analyzer, the charge density of PLGA NPs found to be − 0.753 Mv (Fig. 1C) and the charge dropped to − 0.402 Mv (Fig. 1D) upon loading of tadalafil into PLGA.

### Effect of furosemide and tadalafil on water intake and urinary output

Table 1 shows that water intake of the adenine treated rats (+ ve control) was significantly higher than that of control rats (−ve control). In addition, the water intake was significantly higher in rat treated with chitosan and PLGA NPs compared to control group (−ve control). However, there was no significance between adenine-induced rats (+ ve control) compared to rats pretreated with chitosan and PLGA NPs. In addition, in the first week of treatment there were no differences between drugs_ treated groups either in conventional and NP forms or their combinations compared to adenine-induced rats (+ ve control). However, in the fourth week the water intake in the furosemide NPs and tadalafil NPs treated rats was significantly (*p* < 0.01) higher than the conventional drug treated rats.

The urine volume of the adenine treated rats (+ ve control) was significantly decreased compare to the control group, while there was no significant differences between the control group (−ve control) and rats pretreated with chitosan and PLGA NPs. In the first week of treatment, there were no differences in the urine volume in drugs-treated groups in conventional forms and their combinations compared to the adenine-induced CRF rats (+ ve control), while the urine volume in drug NPs and their combinations was significantly (*p* < 0.001) higher than the adenine-induced CRF rats (+ ve control). In the fourth week of treatment, the urine volume in drugs-treated groups in both conventional, NP forms and their combinations was significantly (*p* < 0.01) higher compared to the adenine-induced CRF rats (+ ve control). In addition, the urine volume in furosemide NPs pretreated rats was significantly (*p* < 0.05) higher compared to furosemide pretreated rats.

### Effect of furosemide, tadalafil and their nanoparticle forms on urine albumin, glucose level and ketone bodies in CRF-induced rats.

**Fig. 3:**
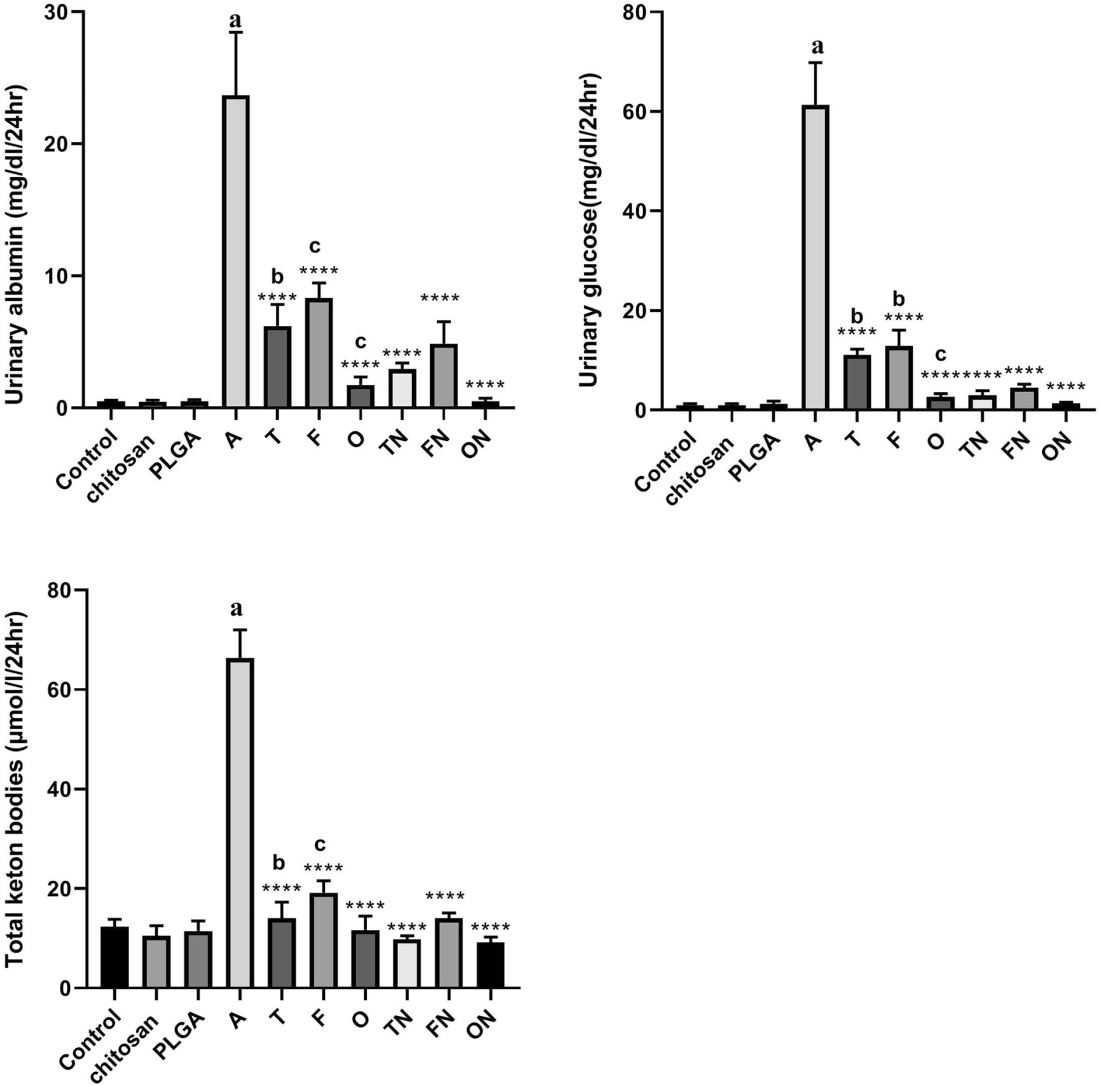
Effect of furosemide (F) and tadalafil (T) in their conventional, nanoparticle (N) forms and their combinations (o) on the levels of glucose, albumin and total ketone bodies in rat’s urine in adenine (A)-induced chronic renal failure. Data are the means ± SEM (*n* = 6). ****p < 0.0001 as compared to the adenine-treated group. ^a^*p* < 0.0001 as compared with the control group. ^b^ *p* < 0.001 as compared with corresponding nanoparticle group (unpaired *t* test). ^c^ *p* < 0.05 as compared with corresponding nanoparticle group (unpaired t test)

CRF-induced rats showed an increase *(p* < 0*.*0001) in urinary albumin, glucose level and ketone bodies compared to negative control group. There were no significant differences in chitosan and PLGA treated rats compared to control group. Conventional and NPs of tadalafil, furosemide and their combinations showed a significant decrease *(p* < 0*.*0001) in urinary albumin, glucose level and ketone bodies compared to CRF-induced animals. In addition, each of tadalafil NPs, furosemide NPs and furosemide–tadalafil NPs combination showed a significant decrease *(p* < 0*.* 05) in the studied parameters compared to their corresponding conventional drug-treated rats as depicted in Fig. 3.

### Effect of furosemide, tadalafil and their nanoparticle forms on urine osmolarity level and specific gravity in CRF-induced rats

**Fig. 4:**
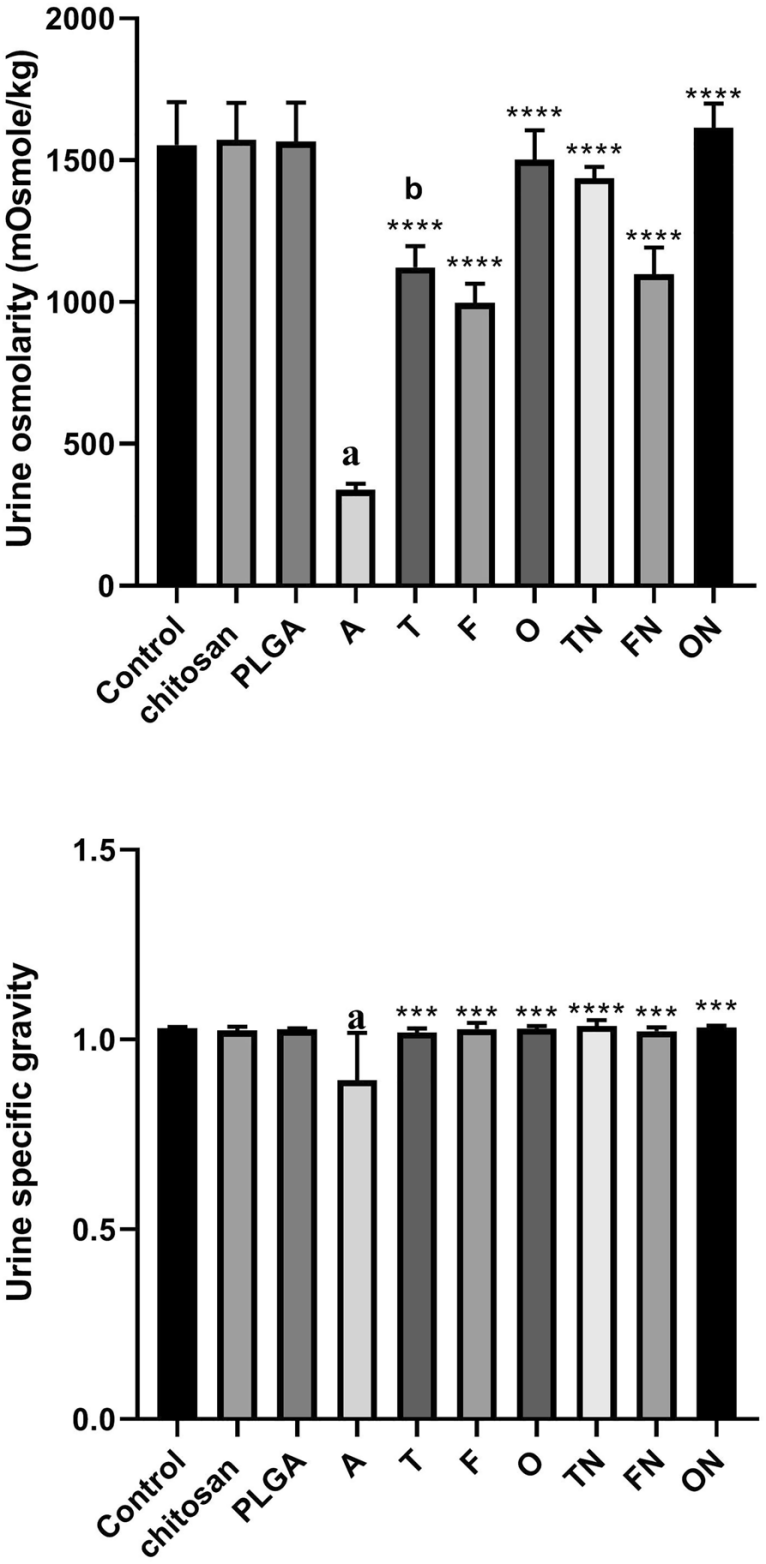
Effect of furosemide (F) and tadalafil (T) in their conventional, nanoparticle (N) forms and their combinations (o) on the urine osmolarity and specific gravity in rats in adenine (A)-induced chronic renal failure. Data are the means ± SEM (*n* = 6). ****p < 0.0001 as compared to the adenine group. ^a^*p* < 0.0001 as compared with the control group. ^b^*p* < 0.0001 as compared with corresponding nanoparticle group (unpaired *t* test)

CRF-induced rats exhibited a significant decrease (*p* < 0.0001) in urine osmolarity and specific gravity compared to negative control group. There were no significant differences in chitosan and PLGA treated rats compared to negative control group. Treatment of CRF-induced rats with conventional or NPs tadalafil, furosemide and their combinations showed a significant increase (*p* < 0.0001) in urine osmolarity and specific gravity. The NP forms of tadalafil showed an increase in urine osmolarity compared to their corresponding conventional drug-treated rats (Fig. 4).

### Effect of furosemide, tadalafil and their nanoparticle forms on serum creatinine level, urea level and total protein in CRF-induced rats.

**Fig. 5:**
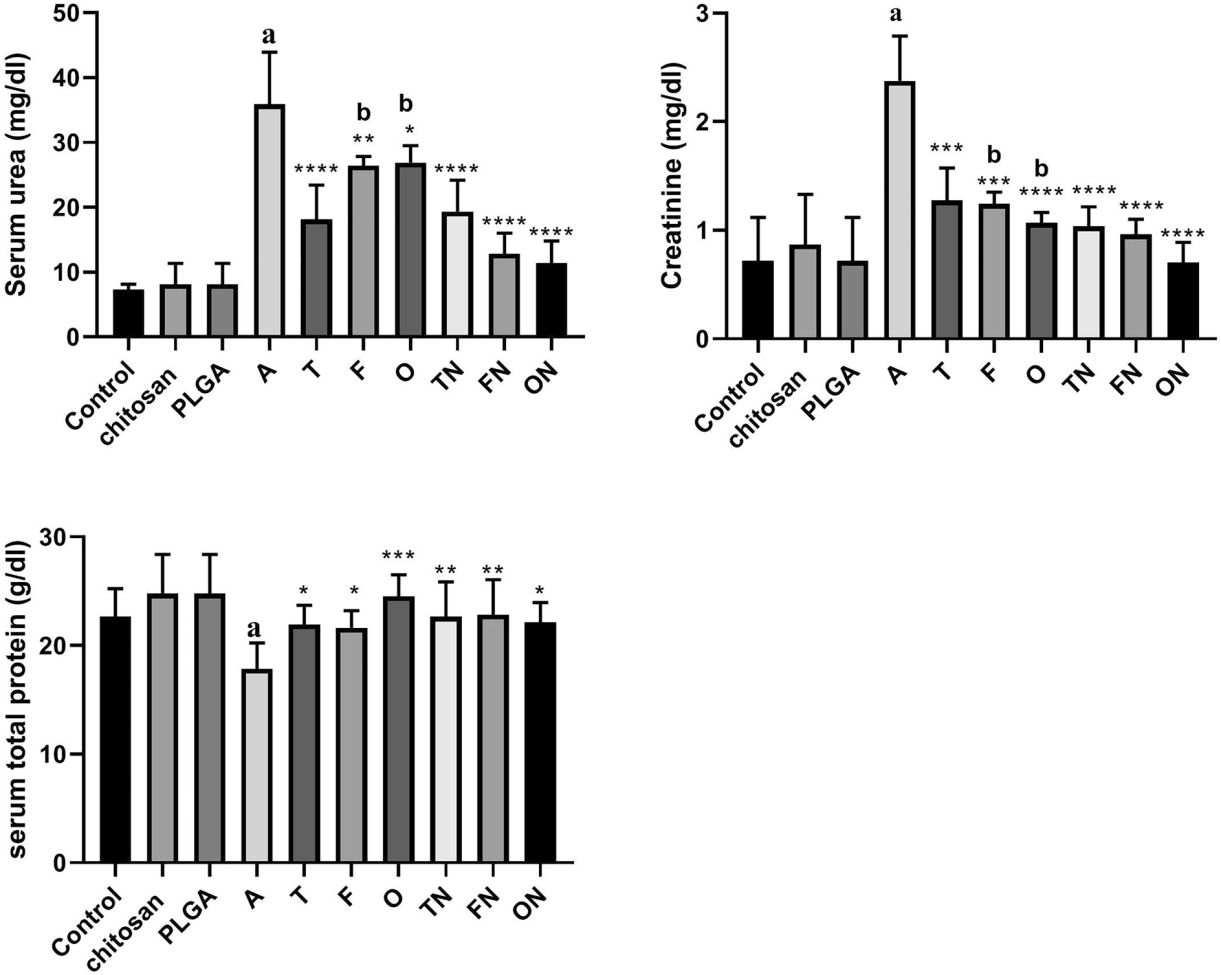
Effect of furosemide (F) and tadalafil (T) in their conventional, nanoparticle (N) forms and their combinations (o) on the levels of creatinine, urea and total protein in rats serum in adenine (A)-induced chronic renal failure. Data are the means ± SEM (*n* = 6). ****p < 0.0001, ***p < 0.001, **p < 0.01 and *p < 0.05 as compared to the adenine group. ^a^*p* < 0.0001 as compared with the control group. ^b^*p* < 0.05 as compared with corresponding nanoparticle group (unpaired t test)

CRF-induced rats showed significant (*p* < 0.0001) increase in serum creatinine and blood urea nitrogen level, whereas a marked decrease was noted in total protein compared to negative control group. However, there were no significant differences in chitosan and PLGA treated rats. Conventional and NPs tadalafil, furosemide and their combinations showed a significant decrease (*p* < 0.05) in serum creatinine and urea levels, while an increase (*p* < 0.05) in total protein compared to CRF-induced rats. A greater reduction in creatinine and urea was observed instead of total protein in CRF-induced rats treated with NP forms particularly with furosemide and the combination (Fig. 5).

### Effect of furosemide, tadalafil and their nanoparticle forms on tissue malondialdehyde, nitrite and glutathione levels in CRF-induced rats

**Fig. 6:**
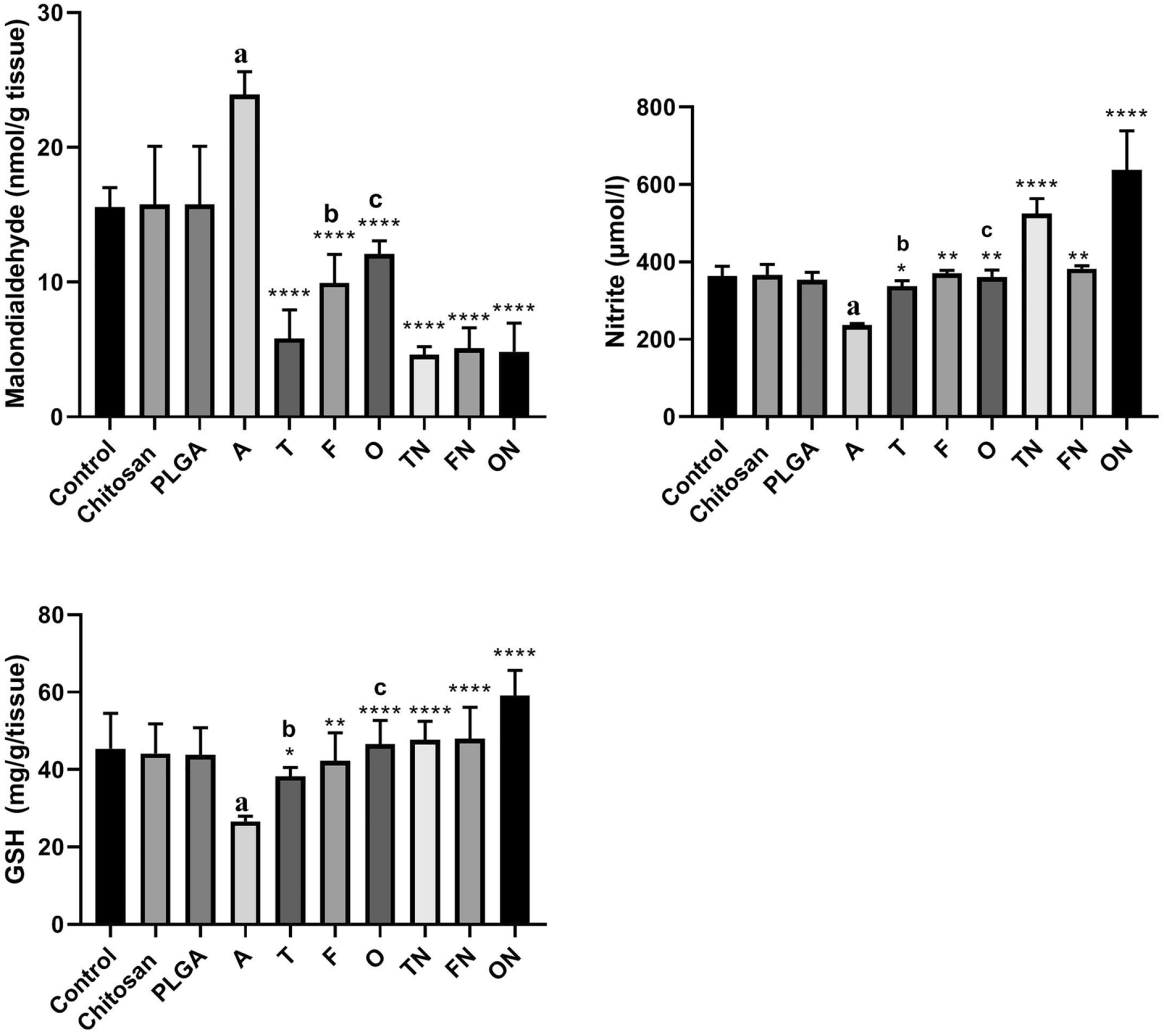
Effect of furosemide (F) and tadalafil (T) in their conventional, nanoparticle (N) forms and their combinations (o) on the levels of malondialdehyde, nitrite and reduced glutathione (GSH) in rat’s kidney tissue in adenine (A)-induced chronic renal failure. Data are the means ± SEM (*n* = 6). ****p < 0.0001, ***p < 0.001, **p < 0.01 and *p < 0.05 as compared to the adenine group. ^a^*p* < 0.01 as compared with the control group. ^b^*p* < 0.01as compared with corresponding nanoparticle group (unpaired *t* test). ^c^*p* < 0.01 as compared with corresponding nanoparticle group (unpaired t test)

As shown in Fig. 6, CRF-induced rats showed significant increase (*p* < 0.01) in tissue malondialdehyde but rather a reduction in nitrite (*p* < 0.0001) and glutathione (*p* < 0.05) levels compared to negative control group. However, there were no significant differences in chitosan and PLGA treated rats compared to negative control group. Conventional and NPs forms of tadalafil, furosemide and their combinations showed a significant decrease (*p* < 0.001) in tissue malondialdehyde, while an increase in nitrite (*p* < 0.0001) and glutathione (*p* < 0.05) levels compared to CRF positive control group. Treatment of ARF-induced rats with furosemide NPs and furosemide–tadalafil NPs combination showed a significant decrease (*p* < 0.01) in tissue malondialdehyde and a marked increase in tissue nitrite and glutathione (*p* < 0.05) particularly with tadalafil NPs and furosemide–tadalafil NPs combination compared to their corresponding conventional drugs treated rats.

### Effect of furosemide, tadalafil and their nanoparticle forms on tissue kidney injury molecule-1 (KIM-1) and neutrophil gelatinase-associated lipocalin (NGAL) levels in CRF-induced rats

**Fig. 7:**
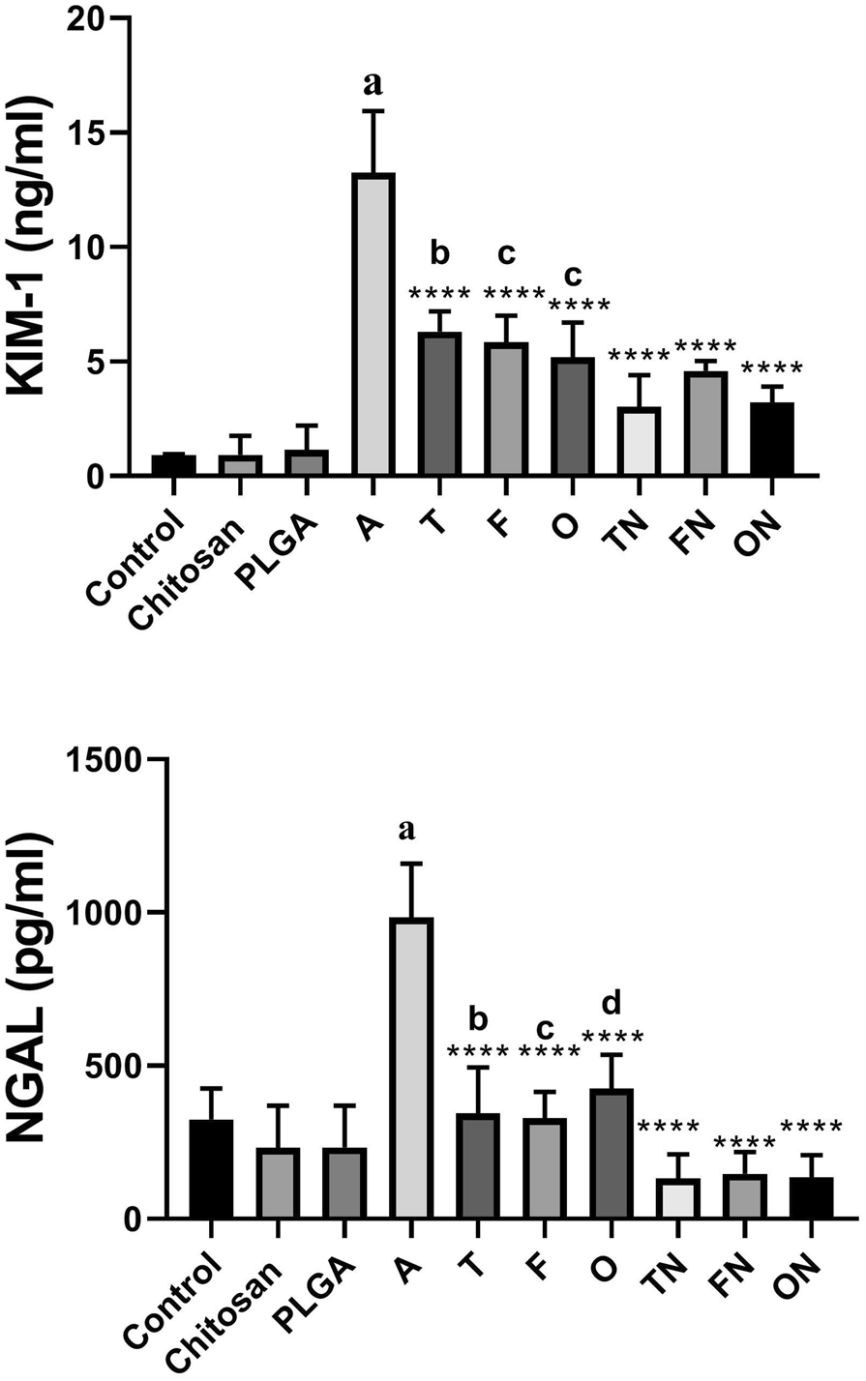
Effect of furosemide (F) and tadalafil (T) in their conventional, nanoparticle (N) forms and their combinations (o) on the levels of kidney injury molecule 1 (KIM-1) and neutrophil gelatinase-associated lipocalin (NGAL) in rats kidney tissue in adenine (A)-induced chronic renal failure. Data are the means ± SEM (*n* = 6). ****p < 0.0001 as compared to the chronic group. ^a^*p* < 0.0001 as compared with the control group. ^b^*p* < 0.05 as compared with corresponding nanoparticle group (unpaired *t* test). ^c^*p* < 0.05 as compared with corresponding nanoparticle group (unpaired *t* test). ^d^*p* < 0.05 as compared with corresponding nanoparticle group (unpaired *t* test)

Adenine–CRF-induced treated rats showed a marked increase in tissue KIM-1 (*p* < 0.0001) and NGAL (*p* < 0.001) compared to negative control group. However, there were no significant differences in chitosan and PLGA treated rats compared to control group. Conventional and NFs forms of tadalafil, furosemide and their combinations showed a significant decrease (*p* < 0.0001) in tissue KIM-1 and NGAL compared to CRF-induced group. Of note, tadalafil NPs, furosemide NPs and furosemide–tadalafil NPs combination showed a significant decrease (*p* < 0.01) in tissue KIM-1 and NGAL compared to their corresponding conventional drug-treated rats as depicted in Fig. 7.

### Histopathological changes in CRF-induced rat model

**Fig. 8:**
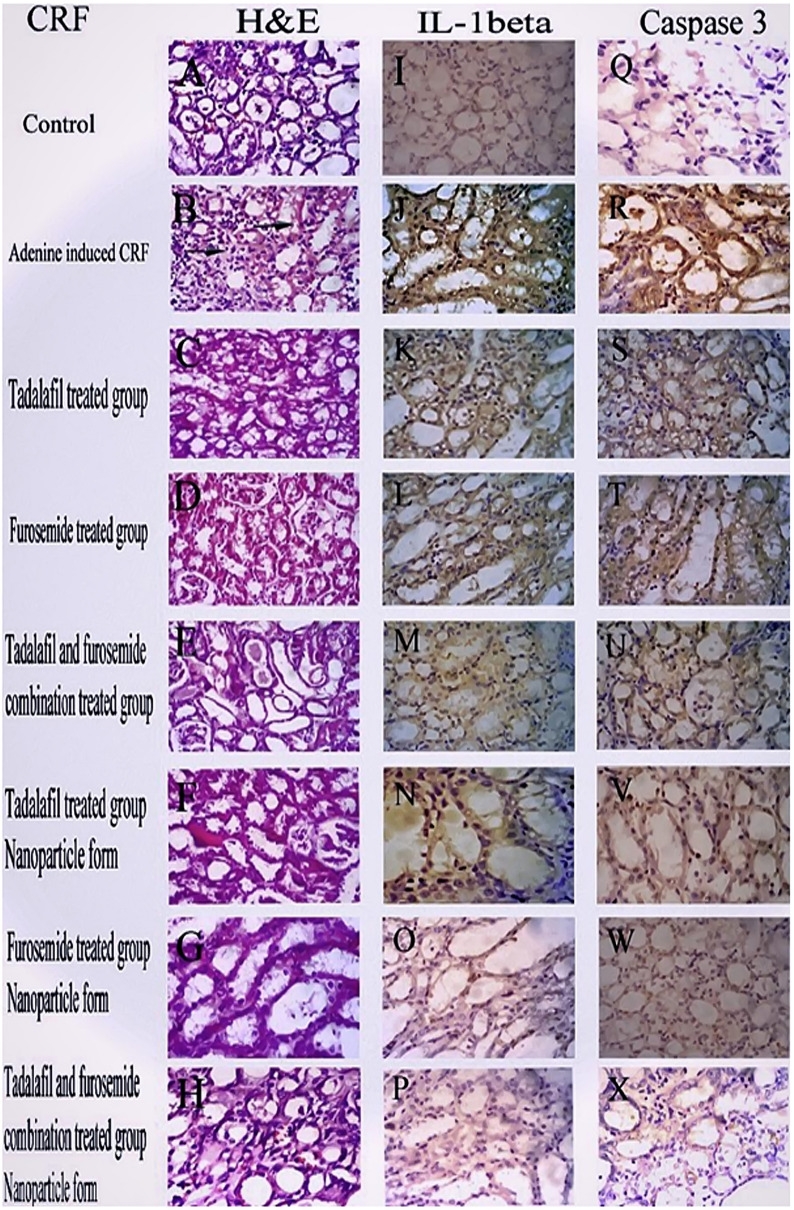
Effect of furosemide and tadalafil in their conventional, nanoparticle forms and their combinations on the histopathological changes as represented in sections (**A**–**H**) and immunoreactivity of both IL-1β sections (**I**–**P**) and caspase-3 sections (**Q**–**X**) in rats kidney tissue in adenine-induced chronic renal failure (CRF)

Figure 8 represents H&E staining of kidney specimens taken from different treated groups. Control groups (section A) show normal histology of renal tissues. In the stained sections of CRF-induced group (section B), tubular necrosis, tubular dilatation and lymphocytic infiltration were observed. H&E staining of CRF-induced animals and treated with tadalafil, furosemide and their combination (sections C, D and E, respectively) showed mild histopathological damage, while NP forms of tadalafil, furosemide and their combination (sections F, G and H, respectively) showed a significantly protection against the severity of renal damage. Thus, the tissue damage score in the NP-treated groups was lower than that of the conventional drugs-treated groups.

### Immunohistochemistry changes in caspase-3 and interleukin-1 beta (IL-1β) expression in CRF-induced rats

Caspase-3 and IL-1β immunoreactivity results are presented in Fig. 8. In the kidney tissues of the control groups, relatively few active caspase-3 (section Q) and IL-1β (section I) positive tubular epithelial cells were detected and. CRF-induced group showed a significant increase in immunoexpression of caspase-3 (section R) and IL-1β (section J). In animals treated with conventional tadalafil, furosemide and their combination in CRF-induced rats showed moderate caspase-3 (sections S, T and U, respectively) and IL-1β (sections K, L and M, respectively) immunoexpression. Interestingly, nanoparticle forms of individual and combinatory drugs in treatment of CRF-induced rats showed more decrease in immunoexpression of caspase-3 (sections V, W and X, respectively) and IL-1β (sections N, O and P, respectively). Accordingly, the immunoreactivity scores of caspase-3 and IL-1β in the NP-treated groups were lower than the conventional drugs-treated groups.

## Discussion

Chronic renal failure (CRF) is a progressive, irreversible process, with uncertain exact aetiology, but diabetes is the most common cause in those starting dialysis [45]. Hypertension, glomerulonephritis and pyelonephritis are less frequent causes. The presence of CRF is associated with an increased mortality risk [46]. In the present study, CRF was induced using adenine oral model. These results showed that water intake, urinary albumin, urinary glucose and total ketones were significantly elevated, while urinary output, urine osmolarity and urine specific gravity were significantly decreased in adenine-induced CRF rats. The results are inconsistent with Rahman et al. who reported elevation in proteinuria and water intake [47]. In addition, Ali et al. reported decreased urine osmolarity; Cho et al. reported decreased in specific gravity and Dos Santos et al. reported decreased in urine output in CRF [48–50].

In present study, the effect of tadalafil (5 mg/kg, p.o.), furosemide (20 mg/kg, i.m.) and their combination either in conventional or NP forms for 28 days to prevent the development of CRF in adenine-induced CRF model in rats. Results showed that water intake, urinary albumin, urinary glucose and total ketones were significantly decreased, while urinary output, urine osmolarity and urine specific gravity were significantly elevated by administration of the previously mentioned drugs which means that the value of these drugs in prevention of the development of CRF in adenine model. Higher enhancement was found with nanoparticulation compared to their conventional forms and could be explained by improved pharmacokinetic properties and enhanced the target ability and bioavailability with the use of drugs nanoforms.

Dysfunction of both protein filtration and reabsorption processes, caused by glomerular injury and tubular impairment associated with CRF may end in increased urinary excretion of albumin and glucose [51, 52]. In addition, as a result of renal ischemia, kidney cortex and medulla shared a somewhat persistent elevation in free fatty acids and the ketone bodies [53].

On the same way, Tomita et al. (2020) reported that tadalafil could enhance protection of the glomerular structures against fibrosis. Tadalafil at low dose 1 mg/kg and high dose 10 mg/kg attenuated proteinuria caused by glomerular injury, diminished glomerulosclerosis, and maintained glomerular structure at chronic kidney disease rat model which is consistent with our results. The only difference is the different model of induction of CRF and different doses [11]. In addition, our results could be supported by the opinion of Rodragguez-Iturbe et al. who mentioned that current available PDE-5 inhibitors have potential clinical value in the treatment of chronic renal disease [54].

The elevation of creatinine, blood urea nitrogen as significant markers of renal injury in that the creatinine level depends on the glomerular filtration rate [55]. Renal dysfunction decrease the ability of creatinine filtration resulting in higher creatinine levels and the GFR is considered to have been halved when creatinine levels twice over the normal value [56]. Elevated blood urea nitrogen is linked either with an increase protein catabolism or conversion of ammonia to urea as a result of the increased synthesis of enzymes involved in urea production [57].

In present study, we found that serum creatinine and blood urea nitrogen were significantly elevated, while blood total proteins was significantly decreased in adenine-induced CRF. These findings are in consistent with the study of Li et al. who reported that adenine treatment led to a significant elevation of the serum creatinine and blood urea nitrogen levels in rats [58]. In addition, in present study, the use of the investigated drugs significantly decreased blood urea and serum creatinine levels, while increased blood total protein levels in adenine-induced CRF. These results are considered as good markers in the improvement of CRF.

Reactive oxygen species plays an important role in the pathogenesis of CRF. The free oxygen radicals can cause lipid peroxidation of cellular membrane which lead to renal tubular necrosis [59]. Thus, lipid peroxidation was an important causal factor to the development of kidney damage in the present study. In addition, intracellular reduced glutathione, the main non-enzymatic antioxidant involved in scavenging of free radicals [60], is known to control several cellular functions as gene expression, cell-cycle progression, apoptosis, and metabolism through adjustments of the cellular redox environment [59, 61]. In addition, reduced glutathione appears to be required for nitric oxide synthesis and nitric oxide seemed to be linked with intracellular reduced glutathione through its protection against oxidative reaction of nitric oxide [62, 63]. A state of nitric oxide deficiency secondary to decreased kidney nitric oxide production and/or increased bioinactivation of nitric oxide in CRF [64]. The combination of nitric oxide inactivation by reactive oxygen species and decreased nitric oxide biosynthesis reduce the availability of nitric oxide and that may lead to increased renovascular resistance [65].

In this study, a significant elevation was seen in the tissue malondialdehyde levels and a significant decline in the tissue reduced glutathione and nitrite levels in adenine-induced CRF rats. These findings are consistent with Vaziri et al. who reported that malondialdehyde level was increased, while nitrite levels was decreased in nephrectomy model of CRF [66]. Treatment with tadalafil, furosemide and their combination either in conventional or NP forms in the present study improve these parameters with the superiority of nanoparticulation which could indicate that our drugs may be effective in prevention of CRF. Because of the important protective role that nitric oxide plays in the renal systems, and by tadalafil inactivation of cyclic 3′,5′-guanosine monophosphate, the second messenger for nitric oxide, this may explain the improvement in nitric oxide levels, related anti-oxidants as reduced glutathione and decreased progression of CRF–adenine model. In addition, studies showed that enhanced nitric oxide synthase activity by furosemide could be linked with increased renal plasma flow induced by furosemide [67]. Thus, we explained that nitric oxide-pathway stimulation in the kidney could be one of the mechanisms by which furosemide exerted its anti-oxidant effects.

NGAL is present in several organs; under normal physiological conditions, NGAL remains at a low expression level in the kidneys, trachea and gastrointestinal tract. However, when ischemia occurs, the secretion of NGAL in the thick ascending limb of renal tubules increases rapidly [68]. Moreover, the statement that KIM-1 may serve as a biomarker of kidney injury may be useful in detection and observing of nephrotoxicants [69]. KIM-1 expression is not detectable in normal kidney but is upregulated in renal failure [70]. In addition, other reported that KIM-1 mRNA levels have been shown to elevate more than any other gene after kidney injury [71].

In this study, we reported that NGAL and KIM-1, have been established as indicator of CRF, were significantly elevated in adenine-induced CRF rats. These findings is consistent with previous studies of Ali et al., who reported that NGAL was markedly increased in adenine-induced CRF and probably supported by a clinical study of Sabbisetti et al. who mentioned that KIM-1 could confirmed as biomarker of acute kidney injury and CRF [72, 73]. It also recognized that NGAL is up-regulated during the course of kidney damage and contributes in nephrogenic healing and regeneration [68].

Furthermore, the present study found that chronic treatment with tadalafil, furosemide and their combination, both in conventional and NP forms for 28 days significantly decreased NGAL and KIM-1 levels in adenine-induced CRF nearly to the same levels of the control group. Such treatment with the NP forms of furosemide and tadalafil showed more marked improvement. Thus, the improvement of adenine-induced CRF by the using of the investigated drugs probably explained on the basis of the reduction that occurred in NGAL and KIM-1 levels.

On the same experimental way, the previous histopathological studies in CRF showed that light and electron microscopy after adenine administration showed tubulointerstitial damage with infiltrating leukocytes, interstitial edema and widening of the Bowman᾿s space in adenine treated rats [74]. These results are consistent with the results of our study in histopathological changes in adenine-induced CRF. In addition, we can explained the improvement of CRF by chronic treatment with tadalafil, furosemide and their combination either in conventional or NP forms for 28 days on the basis of improvement of histopathological findings in the present study.

Chronic renal failure is identified to be accompanying with generation of destructive free radicals and with inflammatory actions. Inflammatory cytokines had an important roles in the development and progression of CRF. It is well acknowledged that the levels of numerous inflammatory cytokines were higher in CRF patients, such as tumour necrosis factor-α, IL-6 and IL-1β [75]. In addition, adenine treatment could induce a highly increase in plasma concentrations of some inflammatory cytokines, such as TNF-α and IL-1β [72]. As oxidative stress is directly involved in the pathogenesis of CRF, it can also result in mitochondrial related apoptosis and aggravate renal dysfunction [76]. Therefore, we measured levels of the apoptotic protein, caspase-3. Furthermore, studies reported that a renal indicators of cell death include caspase-3, which is a vital mediator of programmed cell death (apoptosis) is a probable mechanism involved in the adenine-induced CRF. In addition, caspase-3 activation has been reflected as an apoptotic key, and this is essential for the formation of apoptotic bodies and loss of cell function [77].

In agreement with Ali et al. and Priante et al. the immunohistochemical analysis in the present study indicated that protein expression of both interleukin 1β and caspase-3 significantly elevated in renal cortex in adenine-induced CRF [72, 77]. Our findings could give explanation for the improvement of adenine-induced CRF by chronic treatment with tadalafil, furosemide and their combination either in conventional or NP forms for 28 days through reduction of protein expression of both interleukin 1β and caspase-3. NP forms of tadalafil and furosemide produce a more noticeable improvement.

Overall, the biochemical, histopathological and immunohistochemistry results of this study strongly support the renopreventive effect of tadalafil and furosemide in both conventional and nanoparticle forms in adenine-induced CRF. Furosemide–tadalafil combination in their conventional forms showed greater renal improvement compared to individual drugs. Furthermore, nanoparticle forms of drugs showed higher improvement either individually or in combination compared to conventional forms; however, there are no clinically approved nanoparticles that specifically target the kidney for therapeutic or imaging applications. Further studies should be done for the application of nanoparticle-loaded dugs towards kidney diseases.

## Conclusions

These findings demonstrate that the administration of tadalafil, furosemide and their combinations in both conventional and nanoparticle forms improves CRF in adenine-induced rat model particularly with drug combination nanoforms.

## Supplementary Information

**Additional file 1**: Table S1.

**Additional file 2**: Prism file statistics for raw data.

## Data Availability

The data sets used and/or analysed during the current study are available from the corresponding author on reasonable request (Additional files 1, 2).
