# Supplementary material for: Effects of furosemide and tadalafil in both conventional and nanoforms against adenine-induced chronic renal failure in rats
Source: Eur J Med Res. 2022 Jul 11;27:117. doi: 10.1186/s40001-022-00747-3 (PMC9275182; doi:10.1186/s40001-022-00747-3)
Supplement: Supplementary file 1 — Additional file 1: Table S1. [file 40001_2022_747_MOESM1_ESM.docx]

**Table 1. Effect of furosemide (20 mg/kg,i.m.), tadalafil (5mg/kg, p.o.) and its nanoparticles form pretreatment on urine volume and water intake in CRF-induced rat.**

| **Groups** | **Water intake (ml/24 hr) 1st week** | **Water intake (ml/24 hr) 4th week** | **Urine output (ml/24 hr) 1st week** | **Urine output (ml/24 hr) 4th week** |
| --- | --- | --- | --- | --- |
| **-ve Control** | **19.30 ± 1.207** | **18.80 ± 1.214** | **10.15 ± 0.7228** | **9.650 ± 1.075** |
| **Chitosan** | **41.67 ± 3.57ₐ** | **41.67 ± 3.57ₐ** | **10.17 ± 1.229** | **10.17 ± 1.229** |
| **Poly lactic-co-glycolic acid** | **41.67 ± 2.789ₐ** | **41.67 ± 2.789ₐ** | **9.667 ± 0.9458** | **10.17 ± 1.229** |
| **Adenine (+ve control)** | **40.83 ± 8.002ₐ** | **34.5 ± 2.3ₐ** | **2.250± 0.5737ₐ** | **5 ± 1.204 ₐ** |
| **Furosemide (20mg/kg)** | **41.67± 4.944** | **37.00 ± 6.245b** | **2.000± 0.3416 b** | **10.58 ± 0.8796** b** |
| **Furosemide NPs (20**  **mg/kg)** | **39.17± 4.729** | **56.67 ± 4.216** b** | **11.00 ± 1.323**  ****** b** | **14 ± 0.44**** b** |
| **Tadalafil (10 mg/kg)** | **37.50 ± 4.787** | **39.17 ± 2.007 b** | **4 ± 1.138 b** | **10.75 ± 0.5439**** |
| **Tadalafil NPs (10**  **mg/kg)** | **46.67± 3.57** | **48.33 ± 1.667 b** | **12 ± 0.83 **** b** | **12 ± 1.9***** |
| **Furosemide-tadalafil**  **combination** | **48.33± 3.073** | **35.00 ± 3.651** | **4.167± 0.8233 b** | **10.75 ± 1.352***** |
| **Furosemide_ tadalafil**  **NPs combination** | **43.33 ± 2.108** | **48.33 ± 8.724** | **12.92± 1.645**  ****** b** | **12 ± 0.7746**** |

Data are expressed as mean ± SEM*.** p <* 0*.*01, ****p <* 0*.*001 and *****p <* 0*.*0001 as compared with the CRF-induced group (one-way ANOVA followed by Dunnett's multiple comparisons test), ^a^ denotes *p <* 0*.*001 as compared with the control group and ^b^ denotes *p <* 0*.*05 as compared with corresponding nanoparticle group (unpaired *t*-test).
